# Human protein-RNA interaction network is highly stable across mammals

**DOI:** 10.1186/s12864-019-6330-9

**Published:** 2019-12-30

**Authors:** Aarthi Ramakrishnan, Sarath Chandra Janga

**Affiliations:** 1 Department of Biohealth Informatics, School of Informatics and Computing, Indiana University Purdue University, Indianapolis, IN 46202 USA; 2 Centre for Computational Biology and Bioinformatics, Indiana University School of Medicine, Indianapolis, IN 46202 USA; 3 Department of Medical and Molecular Genetics, Indiana University School of Medicine, Indianapolis, IN 46202 USA

**Keywords:** RNA binding proteins, CLIP-seq, Gene regulatory network, Protein-RNA interactions, Network evolution, Post-transcriptional control, Evolution of binding sites, Genotype-phenotype, Gene expression dynamics

## Abstract

**Background:**

RNA-binding proteins (RBPs) are crucial in modulating RNA metabolism in eukaryotes thereby controlling an extensive network of RBP-RNA interactions. Although previous studies on the conservation of RBP targets have been carried out in lower eukaryotes such as yeast, relatively little is known about the extent of conservation of the binding sites of RBPs across mammalian species.

**Results:**

In this study, we employ CLIP-seq datasets for 60 human RBPs and demonstrate that most binding sites for a third of these RBPs are conserved in at least 50% of the studied vertebrate species. Across the studied RBPs, binding sites were found to exhibit a median conservation of 58%, ~ 20% higher than random genomic locations, suggesting a significantly higher preservation of RBP-RNA interaction networks across vertebrates. RBP binding sites were highly conserved across primates with weak conservation profiles in birds and fishes. We also note that phylogenetic relationship between members of an RBP family does not explain the extent of conservation of their binding sites across species. Multivariate analysis to uncover features contributing to differences in the extents of conservation of binding sites across RBPs revealed RBP expression level and number of post-transcriptional targets to be the most prominent factors. Examination of the location of binding sites at the gene level confirmed that binding sites occurring on the 3′ region of a gene are highly conserved across species with 90% of the RBPs exhibiting a significantly higher conservation of binding sites in 3′ regions of a gene than those occurring in the 5′. Gene set enrichment analysis on the extent of conservation of binding sites to identify significantly associated human phenotypes revealed an enrichment for multiple developmental abnormalities.

**Conclusions:**

Our results suggest that binding sites of human RBPs are highly conserved across primates with weak conservation profiles in lower vertebrates and evolutionary relationship between members of an RBP family does not explain the extent of conservation of their binding sites. Expression level and number of targets of an RBP are important factors contributing to the differences in the extent of conservation of binding sites. RBP binding sites on 3′ ends of a gene are the most conserved across species. Phenotypic analysis on the extent of conservation of binding sites revealed the importance of lineage-specific developmental events in post-transcriptional regulatory network evolution.

## Background

Numerous studies over the recent years have woven into them the theory of sequence conservation. Though few studies contest over whether sequence conservation truly suggests an indispensable function [1, 2], most findings suggest that sequences conserved across a large number and diverse range of species have important functions associated with them [3–7], and this stands as the fundamental principle of comparative genomics [8]. Ultraconserved elements have been known to evolve twenty times slower than the rate at which genomic sequences typically do [9], and 23% of such elements have been validated to be protein-coding sequences. Indeed, studies on unicellular organisms revealed that genes coding for essential proteins were more conserved than nonessential ones [7, 10, 11]. Protein coding sequences conserved across multiple species are of great significance since they encode for proteins that are part of indispensable biological functions.

Certain highly conserved protein coding sequences have shown a significant functional enrichment for RNA binding activity and splicing regulation [9], and several existing studies reveal that RNA-binding proteins (RBPs) are highly conserved across species [12–16]. RNA binding proteins (RBPs) associate with specific mRNA sequences [17, 18], and play a key role in splicing, polyadenylation, transportation and localization of mRNA within the cells [12]. In fact, expression levels of RBPs are tightly regulated in normal physiological conditions and their misregulation is associated with disease phenotypes, likely due to alteration in the expression of the corresponding target transcripts [19, 20]. Hence, post-transcriptional networks governed by RNA-binding proteins are vital in maintaining cellular homeostasis.

Previous studies in lower eukaryotes have shown the existence of rewiring of post-transcriptional regulons. Such studies have largely focused on PUF family; one of the most conserved family of regulons across a wide range of species [21]. Studies on the *Saccharomyces* lineage indicate that there has been a considerable amount of functional rewiring of such regulons within known fungal species [22, 23], wherein the same set of regulons have been found to have different functions among diverse fungal clades. Apart from functional rewiring of regulons themselves, a similar phenomenon was observed for the targets of Puf3p. Though Puf3p recognizes identical genomic element across species, proteins encoded by the targets of Puf3p vary from one species to another [24, 25]. This indicates a possible functional rewiring of targets of Puf3p.

Although investigations on the conservation of RBP targets have been carried out in yeast, little is known about the extent of conservation of the binding sites of RBPs across mammalian species [13, 22, 23]. Although sequences of vertebrate genomes have been employed for comparison of genomes and analysis of the process of evolution among species [8], such studies were directed at sequence conservation of constrained elements, CNEs (Conserved Non-coding Elements) and HARs (Human Accelerated Regions). While some studies focused on the conservation of transcription factor binding sites (more specifically, the enhancer regions) among vertebrate species [6, 26, 27], our understanding of the evolution of binding sites of RBPs across the mammalian genomes is rather limited. Here, we investigate the conservation of the binding sites of RBPs across a large number of mammalian species using experimental CLIP-seq datasets - which can provide bonafide recognition elements of the individual RBPs and MAF (Multiple Alignment Format) files - reflecting the evolutionary trajectories of genomic loci, to study the evolutionary dynamics of mammalian post-transcriptional regulatory networks.

## Results

### Overview of the analysis

We obtained BED (Browser Extensible Data) files reflecting the binding peaks of an RBP, resulting from running a unified peak calling framework on more than 60 human CLIP-seq datasets from CLIPdb [28] (see Materials and Methods). Twenty-two Multiple Alignment Format (MAF) files, each corresponding to a human chromosome were also obtained from UCSC Genome Browser [29]. Current MAF files contain the whole-genome alignments of 45 vertebrate species to the human genome stored in a series of blocks. Studying the extent of conservation of binding sites of RBPs using multiple alignments over such a diverse range of species can assist in the identification of phenotypic features conserved across both terrestrial and aquatic vertebrates [6].

**Fig. 1:**
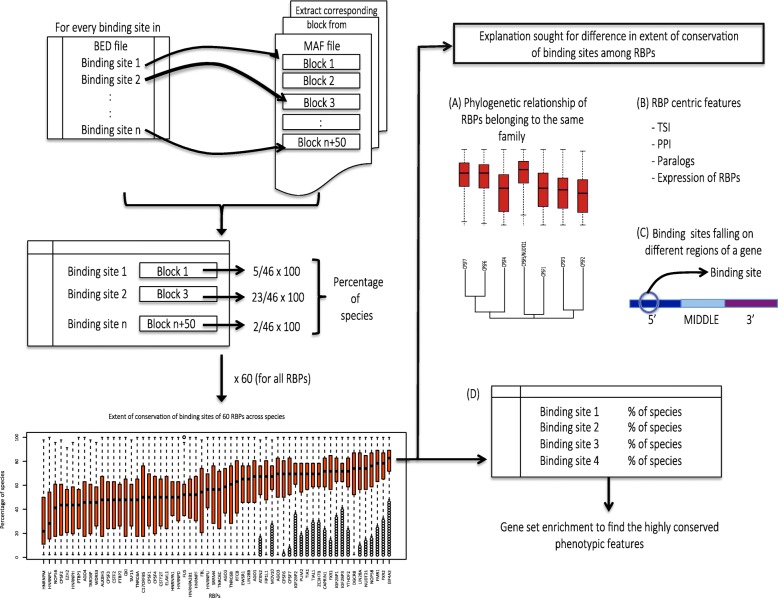
Flowchart showing the various steps employed to study the difference in the extent of conservation of binding sites of RBPs across species. BED files containing binding site coordinates of human RBPs (60 files; one for each RBP) were downloaded from CLIPdb [28]. Multiple Alignment Format (MAF) files (22 files; one for each human chromosome) were downloaded from UCSC genome browser [29], which contain multiple alignments of the whole genomes of 46 vertebrate species arranged in a series of blocks. If the start and end coordinates of a binding site of an RBP from the BED file occurred within the human genome coordinates of a block in a MAF file, the block was extracted. Otherwise, the binding site was ignored. The percentage of species each binding site of an RBP was conserved in was computed from its corresponding MAF block. Repeating this procedure for all RBPs revealed the extent of conservation of binding sites for each RBP. To study the factors contributing to the differences in the extent of conservation of binding sites of RBPs, various RBP-centric and RBP-target level features were examined: A) Phylogenetic relationship of RBPs belonging to the same family, and their extent of conservation of binding sites. B) A multivariate analysis to uncover the RBP-centric features that could influence the extent of conservation of binding sites. C) The extent of conservation of binding sites depending on their location of occurrence along the length of a gene. D) Gene set enrichment to identify the phenotypes associated with an RBP’s post-transcriptional network, ranked by the percentage of species binding sites of an RBP’s target gene were conserved in

**Fig. 2:**
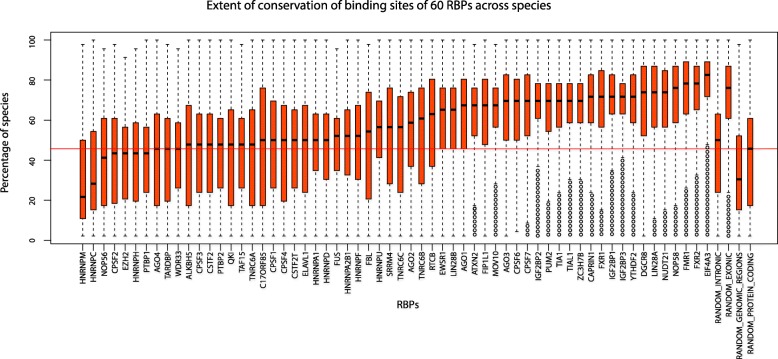
Boxplots showing the extent of conservation of binding sites for each of the 60 human RBPs. Each box plot corresponds to the distribution of the extent of conservation of experimentally identified binding sites of an RBP, across 46 species. Box plots have been arranged in the increasing order of median extent of conservation of binding sites. Circles in the boxplots correspond to the outliers

Using ad hoc python scripts, we processed the BED file of an RBP and twenty-two MAF files simultaneously, to extract the corresponding MAF block of a binding site from a BED file (Fig. 1). If a binding site had no associated MAF block, the binding site was considered to have no conservation across the studied species and was ignored. Once all binding sites of an RBP were mapped to corresponding MAF blocks, the percentage of species that each binding site was conserved in was calculated by counting the number of species in each mapped MAF block (see Materials and Methods). This procedure was repeated for binding sites of all RBPs, which revealed a considerable difference in the extent of conservation of binding sites between RBPs (Fig. 2). In order to uncover the possible explanations for this difference, we asked several specific questions in this study including: (A) Do RBPs belonging to the same family exhibit similar extent of conservation of binding sites? (B) Could certain RBP-centric features explain the difference in the extent of conservation of binding sites among different RBPs? (C) Whether binding sites observed on different regions of a gene exhibit varying extents of conservation. (D) Finally, whether specific human phenotypic features [30] are enriched in genes with highly conserved binding sites of RBPs, to uncover potential genotype-phenotype links in the context of post-transcriptional regulatory networks (Fig. 1).

### Majority of the binding sites for a third of the RBPs are conserved in at least 50% of the species

A considerable difference was observed in the extent of conservation of binding sites for the RBPs included in our analysis (Fig. 2). Across the 60 RBPs, we found the median extent of conservation of binding sites of RBPs to be ranging from 22 to 82% of the species. Majority of the binding sites for 40% of the RBPs were conserved in at least 40% of the species, with this percentage increasing to 50% for a third of the RBPs. Overall, the median extent of conservation of binding sites was 58% of the species. While some RBPs’ binding sites such as those recognized by EIF4A3 and FMR1 were conserved across a large percentage of species, recognition elements of HNRNPM and HNRNPC were found to show a very weak extent of conservation across the vertebrates. In particular, though the binding sites of EIF4A3 were conserved across a large percentage of species, most binding sites of HNRNPM exhibited conservation across not more than 50% of the species. EIF4A3, being a subunit of the exon junction complex (EJC), anchors EJC to mRNA and facilitates its translation [31, 32]. As translation is a fundamental biological process observed in living systems, binding sites of EIF4A3 were very likely conserved across large phylogenetic distances. Although binding sites of HNRNPM and HNRNPC were poorly conserved, the RBPs themselves are key players which influence alternative splicing, pre-mRNA processing and other aspects of mRNA metabolism and transport [33]; suggesting that binding sites of RBPs being poorly conserved does not necessarily indicate that such sites are inessential. Moreover, recent findings reveal that lack of conservation of a sequence does not imply lack of function [34, 35]. Therefore, it becomes essential to understand the variation in the extent of conservation of the binding sites with evolutionary distance, to study whether close relatives exhibit high propensity for conservation of binding sites. We also note that although RBPs such as NOP56 and NOP58 arise from the same protein family [36, 37], their binding sites were not found to exhibit similar extents of conservation (*p* < 2.2e^− 16^, Wilcoxon rank sum test); median extent of conservation being 41 and 76% for NOP56 and NOP58 respectively, suggesting a need for a closer examination. Upon comparison of the extent of conservation of binding sites of all RBPs with a random set of binding sites (see Materials and Methods), we found that the median extent of conservation of random protein coding regions (45%) was significantly lower (*p* < 2.2e-16, Wilcoxon rank sum test) than the median extent of conservation of binding sites of AGO2 (58%). AGO2 was used as a reference for comparison against random datasets since its binding site conservation distribution exhibited an intermediate level among the studied RBPs (Fig. 2). We also noted that the median extents of conservation of random intronic regions (50%) and random genomic regions (30%) were significantly lower (*p* < 2.2e-16, Wilcoxon rank sum test) than the median extent of conservation of the binding sites of AGO2, although the median extents of conservation of exonic regions (77%) was significantly higher (p < 2.2e-16, Wilcoxon rank sum test). These observations revealed that the binding sites of RBPs are significantly more conserved than random genomic, random protein coding and random intronic regions of the same length.

### Binding sites of human RBPs are highly conserved across primates with weak conservation profiles in lower vertebrates

**Fig. 3:**
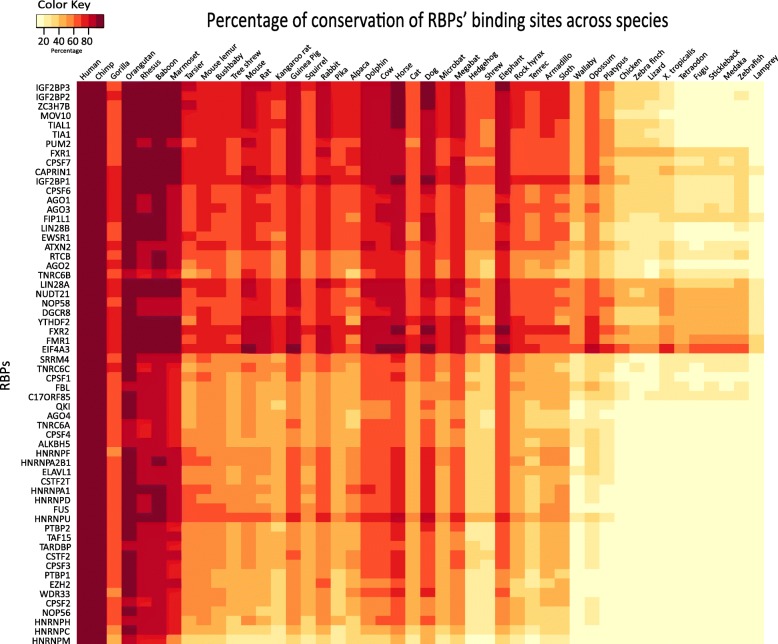
Heatmap showing the conservation of binding sites of RBPs across species. The columns in the heatmap represent species that are arranged based on their evolutionary distances using the R package ape [58], whereas the rows represent RBPs that have been clustered based on Euclidean distance and ward’s method using the function hclust in R. Each cell in the heatmap corresponds to the average extent of conservation of all the binding sites of an RBP in a specific species. While humans and chimps exhibit more than 80% conservation of binding sites of RBPs, lamprey exhibited the least with less than 40% conservation. RBPs NUDT21, EIF4A3 and LIN28A were found to show a high extent of conservation of binding sites across species, whereas the RBPs HNRNPC, HNRNPM and CPSF2 exhibited the least

To ascertain whether the weak conservation of the binding sites of certain RBPs like HNRNPs is due to their loss in selected species versus due to evolutionary distance, binding site conservation profiles of all the RBPs were examined across individual species (Fig. 3). Figure 3 shows a heatmap with the extents of conservation of the binding sites of the RBPs across the 46 species organized by their phylogenetic distance with respect to humans. Upon inspection, each species was found to exhibit a unique extent of conservation of binding sites. More than 80% of the binding sites of RBPs were conserved across most primates, including chimpanzee, orangutan, rhesus macaque, baboon and marmoset. Although gorilla is evolutionarily closer to the aforementioned species, it was not found to exhibit a similar extent of conservation of binding sites as that of the other primates, suggesting a difference in the coding genome of gorilla from humans and other primates [38].

Beyond chicken, binding sites of RBPs such as FUS, EZH2, NOP56, members of HNRNP and PTBP family of RBPs exhibited a poor extent of conservation. To understand whether the loss in the binding sites of these RBPs is due to the loss of these RBPs themselves across distant species, we tested the presence or absence of one-to-one, one-to-many and many-to-many orthologs of human RBPs across the 46 species from Ensembl [39] (Additional file 1: Fig. S1). This analysis unambiguously confirmed our previous observation that RBPs are highly conserved across species [14]. A high conservation of most of these studied RBPs suggested a possible loss or a functional rewiring of the targets of certain RBPs whose binding sites were poorly conserved among birds, lizards and aquatic vertebrates. To investigate whether conservation of binding sites of human RBPs varies with evolutionary distance between species, we compared the overall conservation profile for 37 mammals with the remaining lower vertebrates in our dataset. We found that there is a significantly higher conservation of binding sites in mammals compared to other vertebrates (*p* < 2.2e-16, Wilcoxon rank sum test). In line with our findings, albeit in miRNA post-transcriptional regulatory networks, Chen et al. [40] report that a significant number of miRNA targets are specific to each clade among vertebrates, flies and nematodes.

### Phylogenetic relationship between members of an RBP family does not explain the extent of conservation of their binding sites across species

In an attempt to uncover the factors contributing to the extent of conservation of binding sites, we questioned whether RBPs belonging to the same family exhibit similar conservation profiles of their binding sites. Five distinct RBP families comprising of at least three RBPs each could be identified among the 60 human RBPs that were studied here: AGO, CPSF, HNRNP, IGF2BP and TNRC6 family of RBPs (see Materials and Methods). In order to test whether RBPs belonging to the same family exhibited similar extents of conservation of binding sites across species, matrices corresponding to the evolutionary distances between all pairs of RBPs belonging to the same family were compared to their corresponding matrices generated based on similarity scores of their conservation profiles of binding sites (see Materials and Methods). Briefly, evolutionary distances were calculated based on the phylogenetic tree generated for RBPs belonging to the same family while similarity scores were calculated based on the number of binding sites that exhibited the same extent of conservation among pairs of RBPs.

**Fig. 4:**
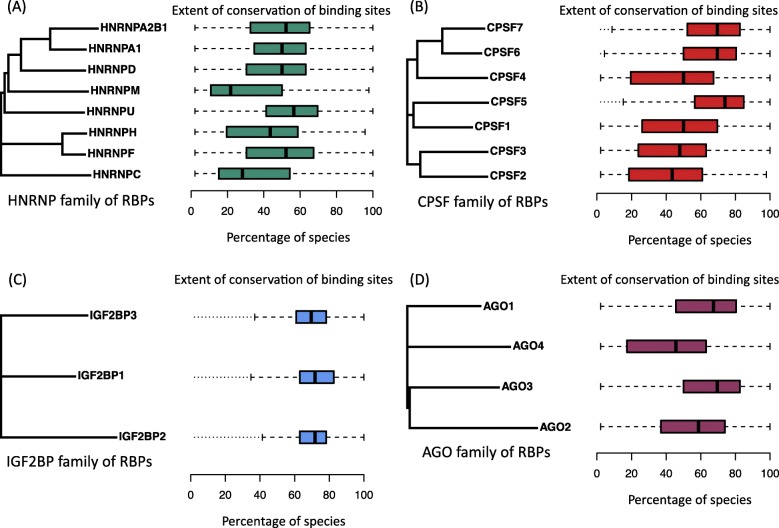
Phylogenetic relationship of RBPs belonging to the same family and their extent of conservation of binding sites across species. RBPs belonging to the same family exhibit varying extents of conservation of binding sites. Box plots represent the extent of conservation of binding sites for the corresponding RBPs in the phylogenetic tree. Comparisons between the phylogenetic trees and the extent of conservation of their binding site profiles, have been shown for members of four RBP families **a** HNRNP family **b** CPSF family **c** IGF2BP family **d** AGO family

Upon computing the evolutionary distances and the similarity scores for all RBP pairs of each family, no association was found between the evolutionary distances and similarity scores (*p* > 0.05, Pearson’s Chi-square test) for any of the RBP families. This analysis confirmed that similarity scores and evolutionary distances were not correlated, suggesting that RBPs belonging to the same family do not exhibit similar extents of conservation of binding sites across species. This observation is also evident from Fig. 4 supporting that phylogenetic relationship of RBPs belonging to the same family is unlikely to be predictive of their extent of conservation of binding sites.

### Expression level and number of targets of RBPs were found to be the most important factors contributing to the differences in the extent of conservation of binding sites

Since RBPs belonging to the same family did not exhibit similar extent of conservation of binding sites, other RBP-centric features that could influence the extent of conservation were explored. This was accomplished by performing a multivariate analysis using a total of 11 RBP-centric features including the number of protein-protein interactions, Tissue Specificity Index (τ), number of RBP-RBP interactions, number of binding sites, length of protein coding transcript of an RBP, median expression of the RBP across tissues at both the protein and transcript level, number of RNA-binding domains, number of paralogs, number of sub-cellular compartments the RBP is documented to be present in and the conservation of an RBP (Table 1). To find the most prominent set of features that could provide an explanation for the variability in the extent of conservation of binding sites between different RBPs, the R package FSelector [41] was employed. The ‘RReliefF’ filter of the FSelector package was utilized because in contrast to other feature selection methods, RReliefF is robust and can handle data with highly interdependent features [42]. The ‘RReliefF’ filter facilitated in fitting the response variable (i.e., median extent of conservation of binding sites of RBPs across species) and the predictor variables (11 RBP-centric features) to generate the attribute importance for each feature (Table 2). Attribute importance refers to the weight assigned to each feature using the algorithm RReliefF (see Materials and Methods). The higher the attribute importance is, the more the impact of the feature in predicting the extent of conservation of binding sites across species for an RBP.

**Table 1:** RBP-centric features employed to uncover the predictor variables likely to explain the variations in the extent of conservation of binding sites for RBPs

Variable	Name of Feature	Description
Response	Median extent of conservation of binding sites of RBPs across species.	For each RBP, the median extent of conservation of binding sites was calculated by computing the median of percentage of species each binding site was conserved in.
Predictors	Tissue Specificity Index (*τ*)	TSI for each RBP was found using the TSI formula as described in a previous study [59] using protein level expression data of RBPs from Human Proteome Map [60].
	Number of binding sites	For each RBP, the total number of binding sites from the BED file that mapped to a block in the MAF file was considered.
	Length of transcript	The length of transcript for each RBP was obtained from Ensembl Biomart [61]. Average of lengths were considered if multiple transcripts were present for an RBP.
	Number of protein-protein interactions	For each RBP, the number of interacting partners was calculated with data obtained from BioGRID [62].
	Median protein level expression of RBPs across tissues	Protein level expression across 17 adult tissues was calculated for each RBP from protein level expression matrix available on Human Proteome Map [60].
	Median transcript level expression of RBPs across tissues	Transcript level expression of RBPs across 16 tissues was calculated using Human BodyMap 2.0 data from Ensembl [39].
	Number of RNA-binding domains	Number of RNA binding domains for each RBP was obtained from a previous study on human RBPs [14].
	Number of Paralogs	The number of paralogs for each RBP was obtained from Ensembl [39].
	Number of sub-cellular compartments	For each RBP, the number of sub-cellular compartments that it is present in was found from UniProt [63].
	Conservation of RBPs	The number of species that each RBP was conserved in was obtained from a previous study [14].
	Number of RBP-RBP interactions	For each RBP, the number of interacting RBPs was computed using data from BioGRID [62].

**Table 2:** Attribute importance from RReliefF feature selection analysis for RBP-centric features described in Table 1

Features	Attribute Importance
Number of binding sites	0.0114
Median protein level expression of RBPs across tissues	0.0048
Number of protein-protein interactions	0.0043
Number of RBP-RBP interactions	0.0026
Median transcript level expression of RBPs across tissues	0.0009
Tissue Specificity Index (*τ*)	0.0005
Number of RNA-binding domains	−0.0025
Length of transcript	−0.0033
Number of sub-cellular compartments	−0.0139
Conservation of RBPs	−0.0145
Number of Paralogs	−0.0195

The most important attributes which were predictive of the extent of conservation of binding sites of RBPs were ‘number of binding sites of an RBP’ followed by ‘median protein expression level of RBPs across tissues’ and the ‘number of protein-protein interactions’. To further investigate whether conservation of RBP targets improves with an increase or decrease in the number of binding sites of RBPs, we carried out a linear regression analysis where we regressed the median extent of conservation of RBP targets with the number of binding sites of RBPs. We observed that increased conservation corresponded to a decrease in the number of binding sites (coefficient = − 1.435, Pr (t) = 1.160e-01) suggesting that RBPs with fewer binding sites are likely to preserve their regulatory interactions across species.

The feature ‘Number of Paralogs of an RBP’ was placed the lowest among all features, suggesting that the number of paralogs of RBPs does not explain the extent of conservation of binding sites; concomitant with the conclusion from the previous section, i.e., RBPs belonging to the same family do not exhibit similar extent of conservation of binding sites. Thousand randomizations of running the RReliefF algorithm using the same set of features did not yield any change in the observed features which are significant. To systematically evaluate whether each of the features contribute significantly towards the extent of conservation, we shuffled the values of each feature across RBPs randomly, one at a time and observed the variation in the ranking reported by RReliefF. Our analysis revealed that shuffling the values of only the top two features namely ‘Number of binding sites’ and ‘Median protein expression level of RBPs across tissues’, resulted in dropping their ranking to the very bottom of the list while other features did not significantly alter their rank, further supporting the importance of these two features in contributing to the extent of conservation.

### Binding sites occurring on 3′ ends of a gene are the most conserved across species

**Fig. 5:**
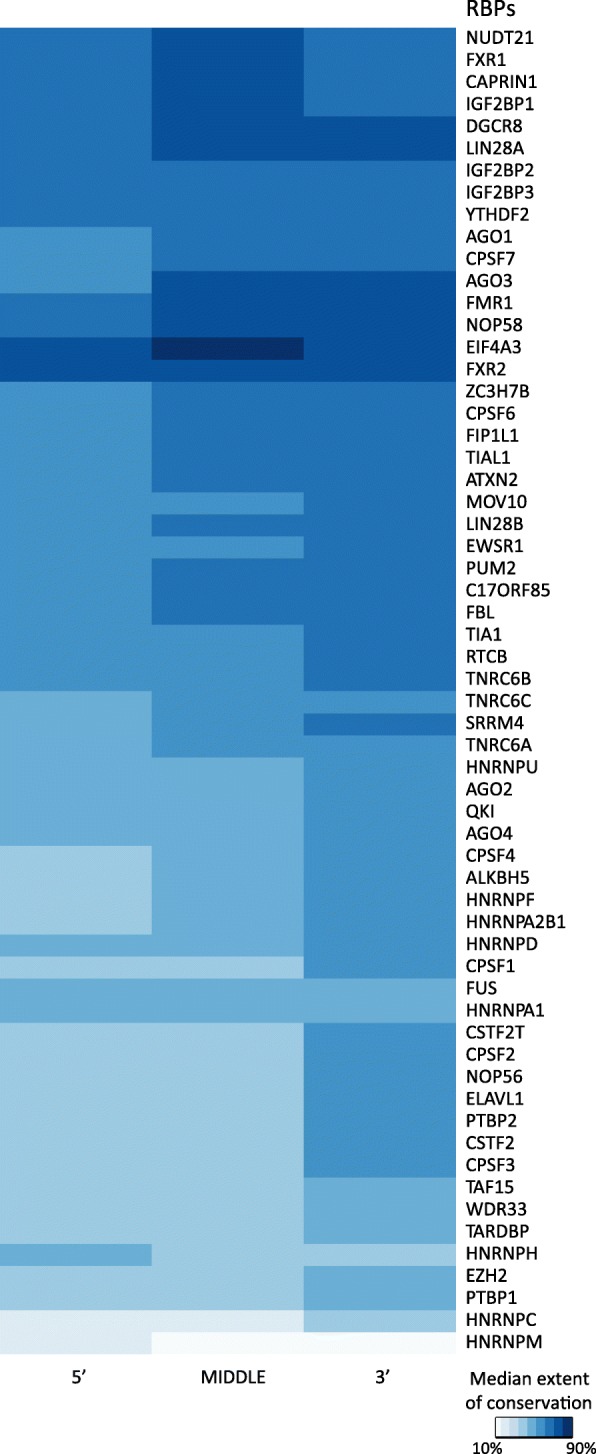
Heatmap showing the extent of conservation of binding sites classified based on their occurrence in the 5′, 3′ or middle region of a gene. Following the classification of all genes in the human genome into 3 equal segments namely 5′, 3′ and middle region, binding sites of RBPs were mapped onto these genic classes. Heatmap shows the median extent of conservation of binding sites of RBPs occurring in the genic classes indicated on the X-axis. Darker colors in the heatmap indicated by the scale bar correspond to higher median extent of conservation of the binding sites

After consideration of potential explanations for the differences in the extent of conservation of binding sites at the RBP-centric level, we sought to investigate whether different regions of a gene exhibit differences in the extent of conservation of experimentally known RBP binding sites. We classified each gene in the human genome into three segments of equal length to define 5′, middle and 3′ regions in the direction of transcription. To study whether binding sites occurring on the 5′, 3′ or the middle region of a gene were more conserved, binding sites of each RBP were mapped onto genes, and the median extent of conservation of binding sites falling on 5′, 3′ and the middle regions were calculated. We observed that 90 % of the RBPs (54/60) significantly (*p* < 0.05, Pairwise Wilcoxon test) exhibited a higher degree of binding site conservation on the 3′ region compared to the 5′ region of a gene (Fig. 5, Additional file 2: Figure S2 and Additional file 3: Figure S3). Sixty-six percent (40/60) of the RBPs were found to exhibit a significantly (p < 0.05, Pairwise Wilcoxon test) higher degree of binding site conservation on the 3′ region compared to the middle region on the gene. These observations indicate that binding sites occurring on the 3′ region of a gene are generally more conserved than the binding sites of RBPs occurring in the rest of the gene. This allows us to suggest that in general, 3′ end regulation processes such as stability control, localization and degradation of transcripts are significantly more conserved post-transcriptional regulatory programs than splicing and translation control of RNA transcripts, although there are certain outliers such as the RBP EIF4A3, which is involved in splicing and translation control of transcripts and exhibited unusually high conservation of its targets. In line with our observations, a previous study on mammalian genes has indicated that there is a selection for 3′ ends in the 3′ UTR of an mRNA [43], and our comparative conservation analysis of RBP binding sites across genic regions reflects this observation.

### Gene set enrichment on the extent of conservation of binding sites reveals phenotypes associated with RBPs

**Fig. 6:**
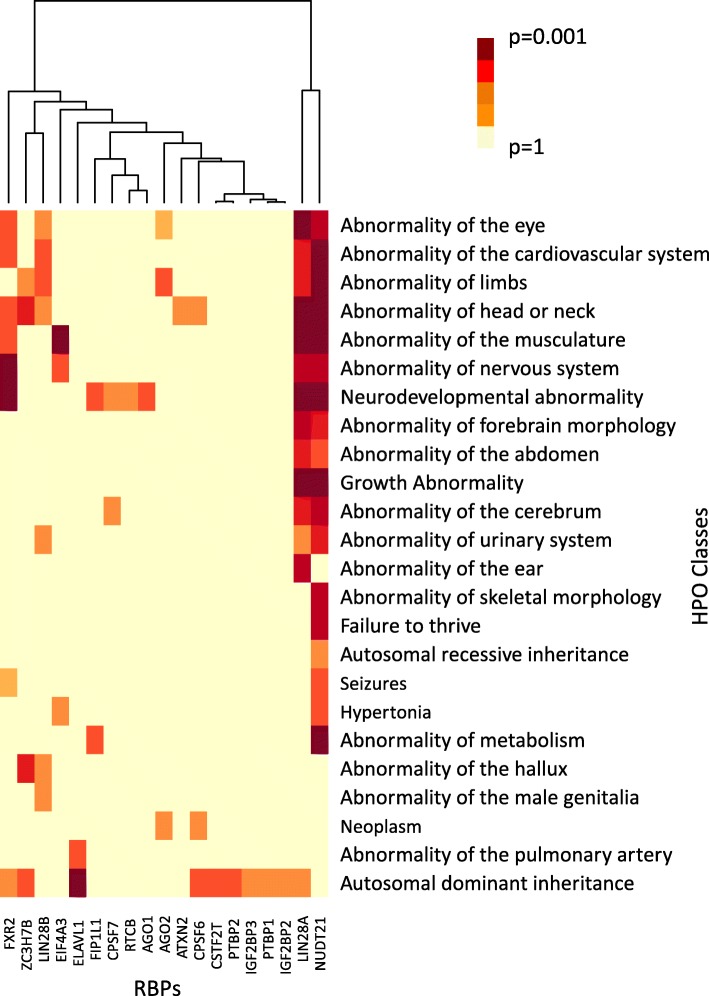
Heatmap showing the Human Phenotype Ontology (HPO) gene sets associated with the binding site conservation profiles of RBPs. Heatmap shows the most significant (corrected *p*-value < 0.05) HPO gene sets that were enriched for genes with highly conserved binding sites. Enriched HPO gene sets were identified by performing a modified gene set enrichment analysis, which uses the extent of conservation of RBP binding sites, as described in the Materials and Methods. Binding sites of NUDT21 yielded the highest number of significant HPO associations

To uncover human phenotypic features associated with highly conserved binding sites of RBPs, gene set enrichment analysis was carried out using the extent of conservation of binding sites of each RBP on a gene as a proxy for its importance, using gene sets from Human Phenotype Ontology [30]. BED files consisting of binding site coordinates were utilized, wherein each binding site was weighted by the percentage of species the binding site was conserved in (see Materials and Methods). This analysis resulted in identifying various gene sets from the Human Phenotype Ontology [30] corresponding to genes with highly conserved binding sites, associated with different RBPs (Fig. 6). These gene sets grouped under a wide range of human phenotypic abnormalities such as ‘Abnormality of the cardiovascular system’, ‘Growth abnormality’ and ‘Neurodevelopmental abnormality’. Out of 19 RBPs that had significant human phenotype ontology gene sets associated to their highly conserved binding sites, 15 of them exhibited a high extent of conservation across species.

The wide range of phenotypes associated with highly conserved binding sites of RBPs indicates that they significantly differ in their phenotypic impact. Studies likewise indicate that RBPs are involved in regulation of a wide range of targets [44]. For instance, highly conserved binding sites of NUDT21 were found to be significantly (*p* < 0.05) involved in ‘Neurodevelopmental abnormality’ (Fig. 6), and literature proposes that NUDT21 is a major player in causing intellectual disability and neuropsychiatric diseases among humans [45]. This suggests that genes involved in neurodevelopmental abnormality may be highly conserved across species. Although binding sites of ELAVL1, CSTF2T, PTBP1 and PTBP2 generally exhibited a poor extent of conservation, their binding sites were significantly associated with various Human Phenotype Ontology gene sets. This finding suggests that lack of conservation of a sequence does not necessarily imply lack of function [34] and supports the notion that RBP networks associated with specific phenotypes might be evolutionarily conserved in specific branches of vertebrates. In particular, we note that several phenotypes associated with neurodevelopmental and cardiovascular processes were significantly enriched for various studied RBPs, suggestive of the modular conservation of their post-transcriptional regulatory networks controlling these phenotypes. As is the case of Human Accelerated Regions [46], several sequences having the same function are altered rapidly among evolutionarily closer species, since the altered version proves beneficial to the species that inherit it. This might be the case for binding sites of RBPs when there are multiple copies of the sites in a gene region or for phenotypes that are not constrained by evolutionary conservation.

## Conclusions

Although sequence conservation has been the focus of several studies in the past, very few studies have focused their attention on the conservation of post-transcriptional protein-RNA interactome. In this study, we dissect the evolutionary dynamics of experimentally known binding sites of 60 human RBPs across 46 vertebrate species to provide a comprehensive understanding of the evolution of these post-transcriptional networks. Our analyses reveals that for most of the studied RBPs, the majority of the binding sites are conserved in at least 40% of the species, suggestive of strong conservation of the binding site profiles. Across all the sites, a median conservation of 58% was found indicating that despite high overall conservation, certain binding sites might be conserved in specific subgroups of species. For instance, the binding sites of HNRNPM were found to be conserved the least, whereas EIF4A3 sites were conserved the most. EIF4A3 is a eukaryotic translation initiation factor belonging to the DEAD box protein family of RNA helicases. Members of this family are implicated in a number of cellular processes involving alteration of RNA secondary structure, such as translation initiation, nuclear and mitochondrial splicing, and ribosome and spliceosome assembly, in addition to the house keeping role of EIF4A3 in facilitating mRNA’s translation [47]. Hence, it is possible to speculate that RBPs involved in core RNA metabolism and translation are likely to exhibit higher conservation of their binding sites compared to core spliceosomal RBPs. However, we cannot dismiss the roles of RBPs whose binding sites are poorly conserved, such as HNRNPM and HNRNPC as they appear to be highly conserved in primates but are increasingly lost in birds and fishes (Fig. 3). These RBPs influence pre-mRNA processing and other aspects of mRNA metabolism and transport suggesting that their functions might be limited to closer phylogenetic distances than translation associated RBPs. We also note that RBPs belonging to the same family based on protein sequence similarity are unlikely to exhibit similar conservation profiles of their binding sites, suggestive of a rapid divergence in the evolutionary trajectories of their post-transcriptional networks. Our observations strongly indicate that RBPs that share the RNA binding domains on their protein chain do not necessarily share the RNA recognition motifs nor are they likely to share the target genes.

A multivariate analysis to uncover the features likely to explain the extent of conservation of post-transcriptional regulatory networks controlled by RBPs supported that the protein expression levels, number of experimentally identified binding sites and protein interaction partners of an RBP significantly contribute to the extent of conservation of binding sites across species. It is interesting to note that in a previous study, RBPs expression and the number of post-transcriptional targets were found to be correlated in the yeast genome [20]. Hence, our observation supports the notion that features that contribute to increasing the plasticity of post-transcriptional networks by increasing the number of targets and/or its diversity across tissues are more likely to contribute to the variations in their extent of conservation.

Analyses to determine differences in the extents of conservation of binding sites across the different genic landmarks, namely 5′, middle and 3′ regions, revealed a significant conservation of binding elements appearing in the 3′ regions. Indeed, 90% of the RBPs exhibited a significantly higher conservation of the binding sites occurring in the 3′ regions of a gene than those occurring in the 5′. These observations allow us to suggest that 3′ end regulation processes such as stability control, localization and degradation of transcripts are significantly more conserved post-transcriptional regulatory processes than splicing and translation control of RNA transcripts. It is possible to speculate from our findings that significant differences in the post-transcriptional network conservation might exist depending on the specific post-transcriptional process controlled by an RBP. Gene set enrichment analysis on the extent of conservation of binding sites to identify the significantly associated human phenotypes revealed an enrichment for multiple developmental processes suggestive of the importance of lineage-specific developmental events in post-transcriptional regulatory network evolution. Although the CLIP-seq datasets used in this study are restricted to human RBPs, with the improvements in the technologies for CLIP-seq protocols and the availability of corresponding CLIP-seq datasets for orthologous RBPs across multiple species, it would be possible to not only study the evolution of the protein-RNA interaction networks from the perspective of multiple mammalian species but to also uncover the patterns of rewiring of post-transcriptional regulatory networks.

## Materials and methods

### Calculation of the percentage of species a binding site of RBP is conserved across

BED files containing the binding sites of 60 RBPs were downloaded from CLIPdb [28]. Each binding site is 20 bp in length. The number of binding sites considered for each RBP is documented in Additional file 4: Table S1. Twenty-two MAF files, one for each chromosome (excluding sex chromosomes), were downloaded from UCSC genome browser [29]. For each RBP, one BED file and twenty-two MAF files were used. The first line of each block in a MAF file consists of human chromosome number, start coordinate and length of the sequence considered. Subsequent lines in the block consist of sequences of vertebrate species that have been aligned to the human genome sequence. BED files for every RBP contain within them binding coordinates of the respective RBPs in humans. Using ad hoc python scripts, if a binding coordinate of an RBP fell within the human chromosomal coordinates of the human genome sequence in the first line of a block in a MAF file, the block was extracted. Binding sites that did not map to any block were excluded. This process was repeated until each binding site of the RBP was considered.

We calculated the percentage of species exhibiting the conservation of a binding site as follows:

$$ \mathrm{P}=\left(\frac{\mathrm{N}}{46}\right)\times 100 $$

where P refers to the percentage of species a binding site was conserved in and N refers to the number of species in the mapped block. Here, the denominator refers to the total number of species for which alignments have been generated. This calculation was made for all binding sites of RBPs.

### Calculation of the extent of conservation of random binding sites

Our analysis revealed that AGO2 exhibits median extent of conservation of binding sites among all RBPs studied here (Fig. 2) and has a total of 162,280 binding sites documented in CLIPdb at the time of this analysis [28]. Therefore, we used it as a reference for comparison and generated 162,280 random intronic, random exonic, random protein coding and random genomic regions; each 20 bp in length. A total of ten random datasets were constructed for each type of random region. A BED file was constructed using the random set of regions and the percentage of species each random region was conserved in, was computed using MAF files. Since there was no significant difference in the distributions among the ten replicates of the random datasets for each region type, one of the datasets was used for showing the representative conservation extents in Fig. 2.

### Evolutionary distances and similarity scores for RBPs belonging to the same family

RBPs were assigned to the same family based on existing literature support. AGO family of proteins comprise of AGO1, AGO2, AGO3 and AGO4 [48]. IGF2BP-family of RBPs includes IGF2BP1, IGF2BP2 and IGF2BP3 [49]. HNRNP proteins comprise of HNRNPA1, HNRNPB1/HNRNPA2B1, HNRNPD, HNRNPM, HNRNPU, HNRNPH, HNRNPF and HNRNPC1/HNRNPC along with other proteins [50]. Among vertebrates, three paralogs of TNRC6 family of RBPs were identified: TNRC6A, TNRC6B and TNRC6C [51]. CPSF1, CPSF2, CPSF3 and CPSF4 are a part of a multiprotein complex, which also include CPSF5, CPSF6 and CPSF7 [52]. For all pairs of RBPs belonging to the same family, 2 parameters were calculated - evolutionary distances and similarity scores (SS).

To calculate the evolutionary distance, Clustal Omega [53] was utilized to generate alignments between each pair of RBPs. These generated alignments were utilized by ClustalW2 – Phylogeny [54] to generate multiple Newick tree formats and the evolutionary distances (ranging from 0 to 1) for each pair of RBPs. The Newick format was also utilized in the construction of phylogenetic trees represented in Fig. 4, using the package phytools [55] in R.

To estimate the similarity between the extents of conservation of binding sites between pairs of RBPs, we calculated the similarity scores for each pairs of RBPs as follows:

$$ \mathrm{SS}=\frac{2\times \mathrm{N}.\mathrm{B}.\mathrm{S}}{\mathrm{N}1+\mathrm{N}2} $$

Where SS refers to the similarity score, N.B.S refers to the number of binding sites conserved in the same % of species, N1 refers to the number of binding sites for the 1st RBP and N2 refers to the number of binding sites for the 2nd RBP.

### The RReliefF algorithm

RReliefF or Regressional ReliefF algorithm uses the probability that two instances belong to two different classes [56]. The probability is modeled with the distance between the values of the target variable of two learning instances [42].

$$ W\left({F}_i\right)=\frac{P_{diff cl\mid diff}{P}_{diff}}{P_{diff cl}}-\frac{\left(1-{P}_{diff cl\mid diff}\right){P}_{diff}}{1-{P}_{diff cl}} $$

In the equation, P_diff_ represents the priori probability that two instances have different feature values and P_diffcl_ represents the prior probability that two instances belong to different classes [42]. The RReliefF algorithm approximates the probabilities, and the feature qualities W [i] are calculated using the above equation [42]..

### Finding human phenotype ontology gene sets associated to highly conserved binding sites

Gene sets refer to groups of relevant genes. In order to find the phenotypic features that pertain to highly conserved binding sites, 7092 Human Phenotype Ontology [30] gene sets were downloaded and converted to GMT (Gene Matrix Transposed) formatted text files. We then used Seten, a previously published method for predicting the phenotypes [57]. Briefly, scores represent the extent of conservation of binding sites. Our approach involved using the binding sites, each weighted by their score, from the input BED file to map onto their corresponding HGNC symbols using a mapping table from Ensembl [39]. After mapping, if multiple scores were available for a gene, median of the scores were taken to represent that gene, which results in a distinct set of genes and their corresponding scores. For every gene set in Human Phenotype Ontology gene set collection, gene set enrichment was performed 1000 times by implementing a permutation based test, where for each gene set, the common genes between the gene set and the input dataset were found and the scores of common genes were compared with the scores of randomly picked genes using Mann-Whitney U test, and a final corrected *p*-value was computed as follows:

$$ \max \left(1-\frac{\#\operatorname{sign}.\mathrm{tests}}{\#\mathrm{total}\ \mathrm{tests}},\frac{1}{\#\mathrm{total}\ \mathrm{tests}}\right) $$

where #sign.tests represents the number of Mann-Whitney U tests significant at *p* < 0.05.

## Supplementary information

**Additional file 1.** Heatmap showing the conservation of RBPs across species. The columns in the heatmap represent species, whereas the rows represent RBPs analyzed in this study for their binding site conservation. Each cell in the heatmap corresponds to the presence of the RBP in the specie.

**Additional file 2.** Boxplots showing the extent of conservation of binding sites occurring in the three genic regions (5’, middle and 3’) for the target genes of each of the 60 human RBPs. Each box plot shows the distribution of the extent of conservation of the binding sites in the three regions (5’, middle and 3’) compared to the overall extent of conservation of the binding sites across all the regions as a reference for a specific RBP. Conservation analyses was performed using experimentally identified binding sites of an RBP across 46 species.

**Additional file 3.** Heatmap showing the relative significance of the extent of conservation of binding sites classified based on their occurrence in the 5’, 3’ or middle region of a gene between pairs of region comparisons. Following the classification of all genes in the human genome into 3 equal segments namely 5’, 3’ and middle region, binding sites of RBPs were mapped onto these genic classes to study their conservation across 46 species. Heatmap shows the significance from Wilcoxon test for pairwise comparison of the different regions for each RBP for their extent of conservation. Darker blue shades correspond to more extreme differences between the compared regions for the extent of conservation of binding sites.

**Additional file 4.** Number of experimentally known binding sites for each of the 60 RBPs employed in this study.

## Data Availability

All the datasets employed in this study are freely available in the cited database resources in the materials and methods section of the manuscript.

## Abbreviations

BED: Browser Extensible Data
CLIP: Crosslinking Immunoprecipitation
EJC: Exon Junction Complex
HPO: Human Phenotype Ontology
MAF: Multiple Alignment Format
PUF: Pumilio and FBF family
RBP: RNA-binding protein
TF: Transcription Factor
