# Supplementary figures and images for: Human protein-RNA interaction network is highly stable across mammals
Source: BMC Genomics. 2019 Dec 30;20(Suppl 12):1004. doi: 10.1186/s12864-019-6330-9 (PMC6936122; doi:10.1186/s12864-019-6330-9)

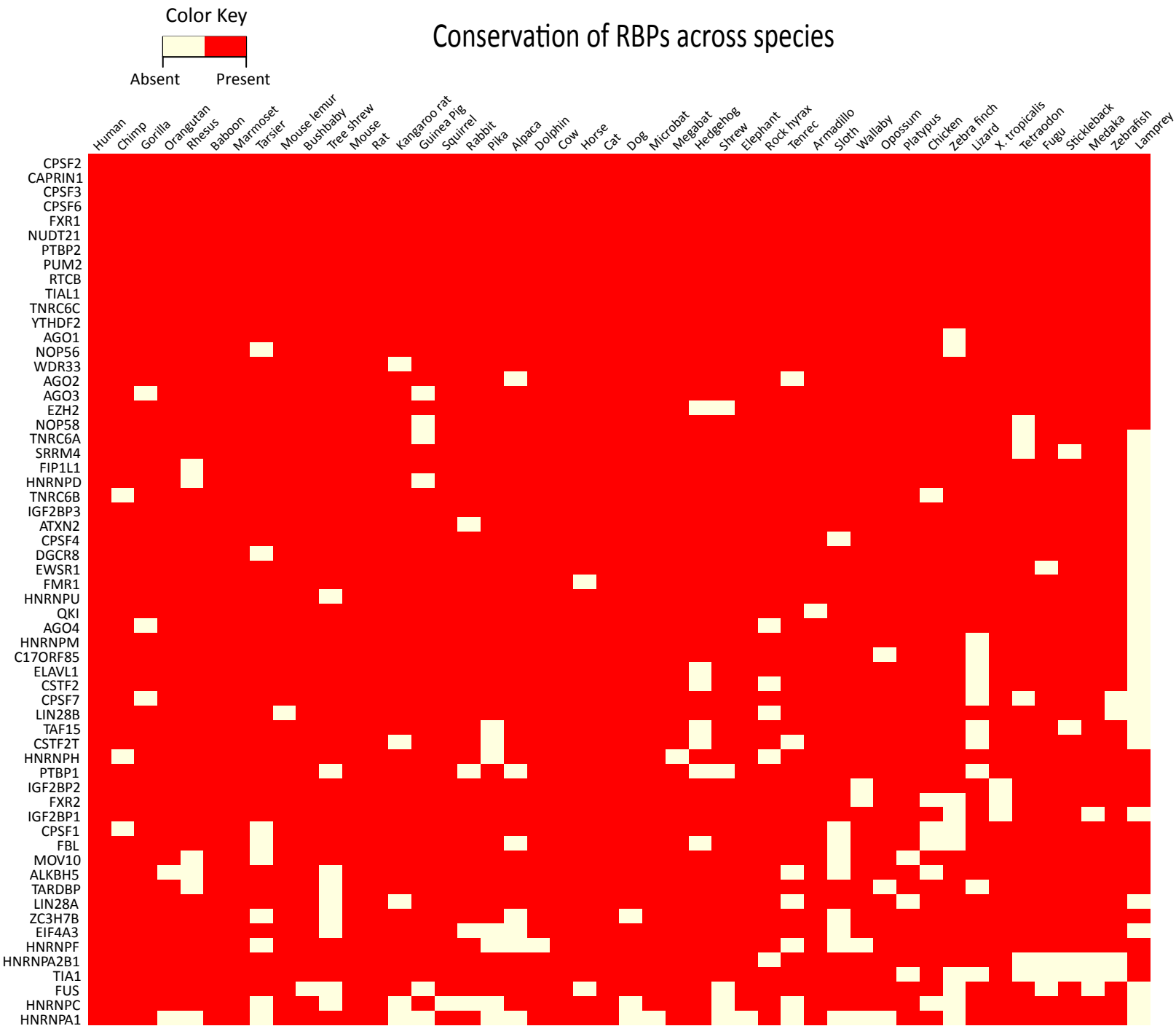

Supplement: Supplementary file 1 — Additional file 1. Heatmap showing the conservation of RBPs across species. The columns in the heatmap represent species, whereas the rows represent RBPs analyzed in this study for their binding site conservation. Each cell in the heatmap corresponds to the presence of the RBP in the specie. [file 12864_2019_6330_MOESM1_ESM.pdf]

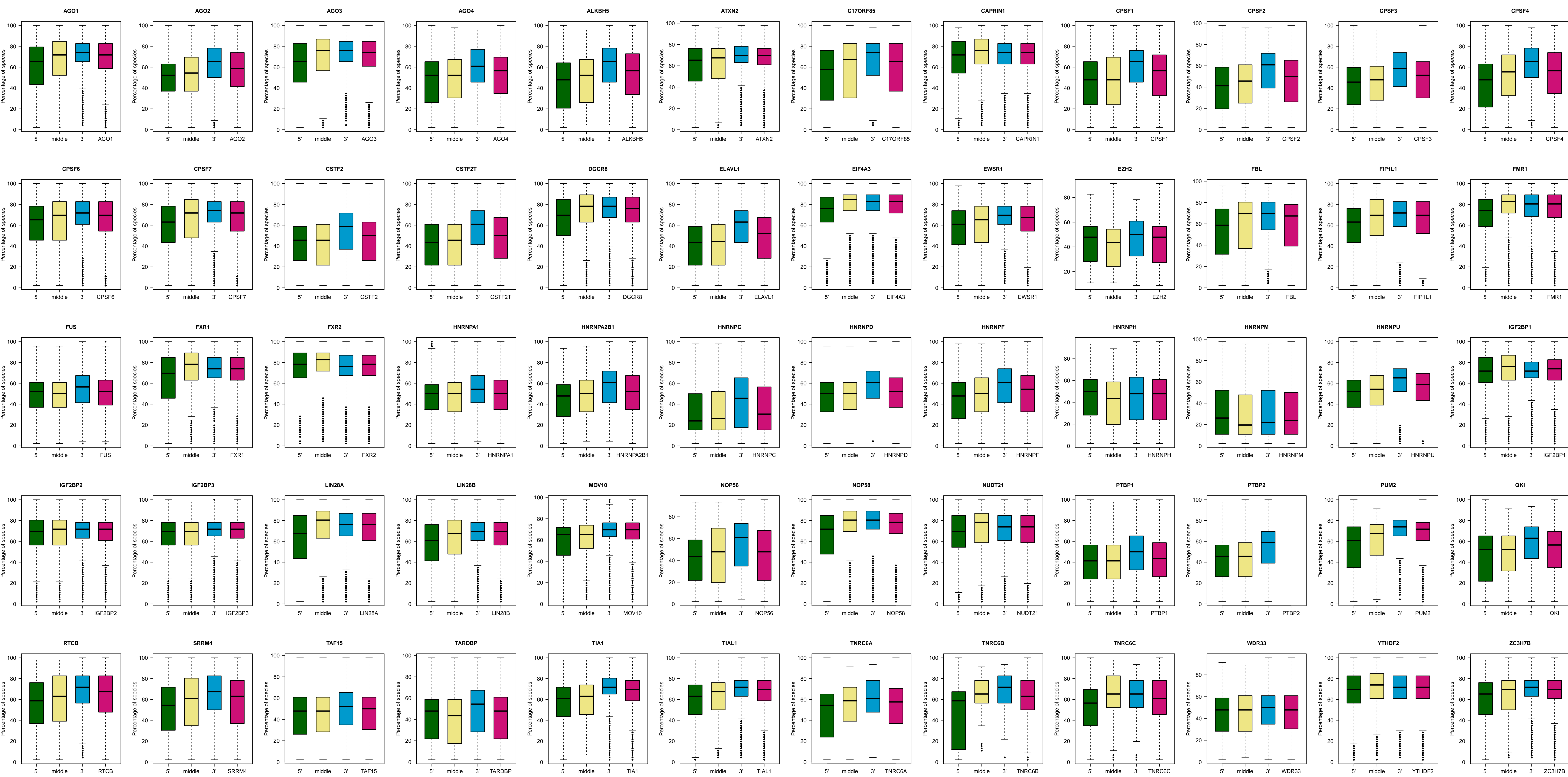

Supplement: Supplementary file 2 — Additional file 2. Boxplots showing the extent of conservation of binding sites occurring in the three genic regions (5’, middle and 3’) for the target genes of each of the 60 human RBPs. Each box plot shows the distribution of the extent of conservation of the binding sites in the three regions (5’, middle and 3’) compared to the overall extent of conservation of the binding sites across all the regions as a reference for a specific RBP. Conservation analyses was performed using experimentally identified binding sites of an RBP across 46 species. [file 12864_2019_6330_MOESM2_ESM.pdf]

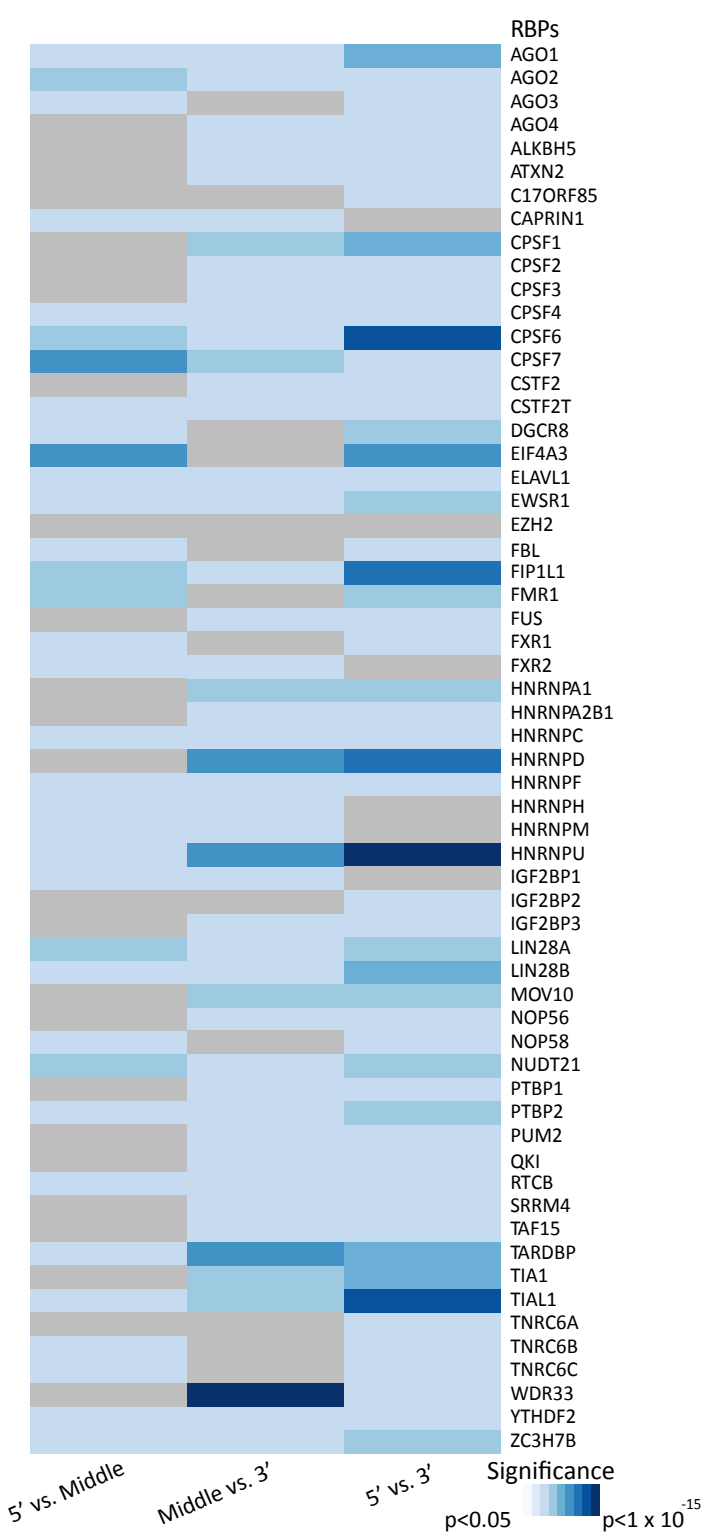

Supplement: Supplementary file 3 — Additional file 3. Heatmap showing the relative significance of the extent of conservation of binding sites classified based on their occurrence in the 5’, 3’ or middle region of a gene between pairs of region comparisons. Following the classification of all genes in the human genome into 3 equal segments namely 5’, 3’ and middle region, binding sites of RBPs were mapped onto these genic classes to study their conservation across 46 species. Heatmap shows the significance from Wilcoxon test for pairwise comparison of the different regions for each RBP for their extent of conservation. Darker blue shades correspond to more extreme differences between the compared regions for the extent of conservation of binding sites. [file 12864_2019_6330_MOESM3_ESM.pdf]
