# Supplementary material for: Human protein-RNA interaction network is highly stable across mammals
Source: BMC Genomics. 2019 Dec 30;20(Suppl 12):1004. doi: 10.1186/s12864-019-6330-9 (PMC6936122; doi:10.1186/s12864-019-6330-9)
Supplement: Supplementary file 4 — Additional file 4. Number of experimentally known binding sites for each of the 60 RBPs employed in this study. [file 12864_2019_6330_MOESM4_ESM.pdf]

RBP	No. of Binding sites
AGO1	33152
AGO2	162280
AGO3	6342
AGO4	1341
ALKBH5	1128
ATXN2	7413
C17ORF85	1048
CAPRIN1	4487
CPSF1	8146
CPSF2	1597
CPSF3	2972
CPSF4	2583
CPSF6	56010
CPSF7	58842
CSTF2	172937
CSTF2T	54321
DGCR8	9228
EIF4A3	23434
ELAVL1	255591
EWSR1	8624
EZH2	269
FBL	2738
FIP1L1	28964
FMR1	9087
FUS	5649
FXR1	2262
FXR2	6905
HNRNPA1	15577
HNRNPA2B1	1783
HNRNPC	99454
HNRNPD	15500
HNRNPF	3892
HNRNPH	4585
HNRNPM	14951
HNRNPU	15287
IGF2BP1	15460
IGF2BP2	9418
IGF2BP3	10276
LIN28A	12728
LIN28B	21611
MOV10	13839
NOP56	2184

NOP58	3720
NUDT21	34619
PTBP1	121226
PTBP2	54243
PUM2	1236
QKI	1168
RTCB	3247
SRRM4	6507
TAF15	4182
TARDBP	32555
TIA1	8590
TIAL1	24197
TNRC6A	659
TNRC6B	367
TNRC6C	586
WDR33	6065
YTHDF2	14379
ZC3H7B	20869
